# Long-term stability of acquired drug resistance and resistance associated mutations in the fungal pathogen *Nakaseomyces glabratus* (*Candida glabrata*)

**DOI:** 10.3389/fcimb.2024.1416509

**Published:** 2024-07-15

**Authors:** Ewa Ksiezopolska, Miquel Àngel Schikora-Tamarit, Juan Carlos Nunez-Rodriguez, Toni Gabaldón

**Affiliations:** ^1^ Department of Life Sciences, Barcelona Supercomputing Centre (BSC-CNS), Barcelona, Spain; ^2^ Department of Mechanisms of Disease, Institute for Research in Biomedicine (IRB Barcelona), The Barcelona Institute of Science and Technology, Barcelona, Spain; ^3^ Catalan Institution for Research and Advanced Studies (ICREA), Barcelona, Spain; ^4^ Department of CIBERinfect, Centro Investigación Biomédica En Red de Enfermedades Infecciosas, Barcelona, Spain

**Keywords:** *Nakaseomyces glabratus* (*Candida glabrata*), drug resistance stability, aneuploidy, mutations, microevolution

## Abstract

The limited number of available antifungal drugs and the increasing number of fungal isolates that show drug or multidrug resistance pose a serious medical threat. Several yeast pathogens, such as *Nakaseomyces glabratus* (*Candida glabrata*), show a remarkable ability to develop drug resistance during treatment through the acquisition of genetic mutations. However, how stable this resistance and the underlying mutations are in non-selective conditions remains poorly characterized. The stability of acquired drug resistance has fundamental implications for our understanding of the appearance and spread of drug-resistant outbreaks and for defining efficient strategies to combat them. Here, we used an *in vitro* evolution approach to assess the stability under optimal growth conditions of resistance phenotypes and resistance-associated mutations that were previously acquired under exposure to antifungals. Our results reveal a remarkable stability of the resistant phenotype and the underlying mutations in a significant number of evolved populations, which conserved their phenotype for at least two months in the absence of drug-selective pressure. We observed a higher stability of anidulafungin resistance over fluconazole resistance, and of resistance-conferring point mutations as compared with aneuploidies. In addition, we detected accumulation of novel mutations in previously altered resistance-associated genes in non-selective conditions, which suggest a possible compensatory role. We conclude that acquired resistance, particularly to anidulafungin, is a long-lasting phenotype, which has important implications for the persistence and propagation of drug-resistant clinical outbreaks.

## 1 Introduction

Although fungi can be part of the natural human microbiome of healthy individuals (Hallen-Adams and Suhr, 2017), they can also be the source of invasive infections that are often fatal in immunocompromised patients (Silva, 2010). Changes related to advances in medical progress such as the extensive use of antibiotics, the aging of the population, or the increased survival of immunocompromised patients have been linked to a growing incidence of fungal diseases (Mason et al., 2012; Gabaldón and Carreté, 2016). Pathogenic yeasts belonging to the polyphyletic genus *Candida* are the most common cause of life-threatening invasive infections as well as of mucosal infections, such as vulvovaginal candidiasis (Gabaldón et al., 2016; Berman and Krysan, 2020).

Antifungal therapy and prophylaxis are key for reducing the mortality and comorbidity associated with fungal infections. However, they are also primary factors driving progressive epidemiological shifts, including diminishing prevalence of *Candida albicans* in favor of non-albicans *Candida* species presenting higher levels of intrinsic and/or acquired resistance, such as *Nakaseomyces glabratus* (*Candida glabrata*) (Lamoth et al., 2018). In addition, recent studies report a growing prevalence of clinical isolates that are resistant to multiple drugs, mostly belonging to non-albicans species such as *N. glabratus* (Beyda et al., 2014; Pham et al., 2014), *Candida kefyr* (Fekkar et al., 2013), *Candida lusitaniae* (Asner et al., 2015), and *Candida auris* (Vallabhaneni et al., 2016). Emergence of drug and multidrug resistance in fungi is particularly worrying given the limited arsenal of antimycotic agents, most of them belonging to one of three major families: azoles, echinocandins, and polyenes (Krysan, 2017). Azoles inhibit ergosterol biosynthesis by binding to one of the enzymes in the pathway (Erg11p), thereby inhibiting cell growth. Polyenes bind directly to ergosterol, which weakens the cell membrane and leads to cell death. Echinocandins block glucan synthase, encoded by *FKS* genes, thereby inhibiting the biosynthesis of β-1,3-d-glucan, a major component of the fungal cell wall (Ksiezopolska and Gabaldón, 2018). Mechanisms of antifungal drug resistance involve alterations in the sequence or expression of the genes encoding the drug targets, overexpression of drug efflux pumps, and gross chromosomal changes (Cowen et al., 2014; Ksiezopolska and Gabaldón, 2018).

Previous studies have shown that gene copy number variations, including whole-chromosome (Chr) aneuploidies, may contribute to antifungal drug resistance (Coste et al., 2006; Selmecki et al., 2006; Sasse et al., 2012; Yang et al., 2013; Anderson et al., 2017; Yang et al., 2017; Todd et al., 2019; Yang et al., 2019). For instance, in *C. albicans*, azole resistance was associated with the presence of an isochromosome (5L) which resulted in two extra copies of the left arm of Chr5 (Selmecki et al., 2006), which carry *ERG11* and *TAC1* (encoding the transcription factor regulating ABC transporter genes *CDR1* and *CDR2*) genes. Furthermore, aneuploidies of Chr3, bearing *CDR1* and *MRR1* (encoding a transcriptional activator of the major facilitator superfamily transporter *MDR1*), and trisomy of Chr7 were connected with increased efflux of the drug (Mount et al., 2018). *N. glabratus* presents a considerable karyotypic variability with many analyzed isolates presenting gross genomic rearrangements, which have been sometimes attributed to a response to antifungal drug treatments (Shin et al., 2007; Muller et al., 2009; Poláková et al., 2009; Healey et al., 2016).

Genomic rearrangements can be advantageous to fungal cells by contributing to rapid responses and adaptation to stress and can represent intermediate evolutionary steps in the acquisition of resistance to unfavorable conditions (Ksiezopolska and Gabaldón, 2018). Supporting this view is the observation that some gross genomic rearrangements, such as aneuploidies, occur at higher rates than specific point mutations, especially under stress conditions (Duesberg et al., 2001; Healey et al., 2016). Hence, they are likely to be the first resistance-conferring alterations that appear spontaneously in an evolving population. Chromosomal aneuploidies result in higher or lower loads of several genes at a time, some of which may be advantageous in specific conditions. However, as they involve the dysregulation of many additional “passenger” genes, they are also expected to have a fitness cost and, consequently, be evolutionarily unstable (Rustchenko, 2007). Considering all this, changes in ploidy can be regarded as a rapidly acquired temporary solution to stress conditions that allows suboptimal survival of the population and facilitates the emergence of fitter, more stable point mutations (Berman, 2016). However, how stable these alterations really are is still poorly investigated.

Persistence of the resistance phenotype has been reported in clinical and *in vitro* studies (Borst et al., 2005; Imbert et al., 2016; Hatwig et al., 2019). Imbert et al. observed loss of resistance to echinocandins (but not azoles) in multidrug-resistant *N. glabratus* after 1 month of treatment discontinuation, and the loss was attributed to the disappearance of an *FKS* mutation. Borst et al. reported the stability of resistance to fluconazole (flz) after 122 days, and Hatwig et al. reported resistance to anidulafungin (ani) and flz after a month of propagation of under no antifungal stress. However, the number of investigated strains was relatively low (one patient, and five and six *in vitro* evolved strains, respectively), and none of the studies included analysis of the genomic changes involved in the emergence or loss of the resistance phenotypes.

In an earlier *in vitro* evolution study (Ksiezopolska et al., 2021), we obtained a collection of strains that successfully adapted to different drug treatment regimes and acquired resistance to one or two drugs. The analysis of their genomes identified newly acquired mutations that are likely to drive the resistance phenotype, including different aneuploidies and point mutations. One unexpected result of that study was that 10 out of 11 mutants carrying chromosomal duplications acquired during flz treatment retained this aneuploidy after 18 subsequent passages in ani, suggesting that aneuploidies are long-lived, at least during exposure to ani. This observation prompted us to investigate the stability of the resistance phenotype and the underlying mutations after propagation under optimal growth conditions. To assess this, we propagated a battery of resistant strains representing a diverse set of resistance mechanisms on optimal growth conditions and subsequently tested the presence of the resistance phenotype and some of the underlying mutations. Our results shed light onto the important question of persistence of genetically acquired resistance against fungal drugs.

## 2 Materials and methods

### 2.1 Strains

The 70 parental strains for this study are mono- and multidrug-resistant strains obtained from directed evolution under drug exposure in a previous study (Ksiezopolska et al., 2021). Detailed information about the strains and their origin (WT strains) can be found in **Supplementary Table S1**. For the ease of the interpretation of the results, we decided to avoid naming the samples in the main text. However, in **Supplementary Tables S2–S4** and **Supplementary Figures S1–S5**, one can find names of the original susceptible WT stain (for example CBS138 or EF1620) and the name of the parental strain in the study (samples that were previously evolved in ani (ANI) and then in flz (AinF), flz (FLZ), and then in ani (FinA), and both drugs are the same time (ANIFLZ), the “_YPD” suffix indicating the propagation in YPD in this study, “_rep” indicating the biological replicate). Please note that some of the ani-evolved parental strains (ANI) belong to MDR as they acquired resistance to both ani and flz. In other words, ANI samples in this table correspond to samples evolved in ani and can be included in ANIR samples which are resistant to ani only or MDR samples which are resistant to both drugs. Aneuploid samples (AS) were obtained from 10 FLZ samples and their 10 direct FinA progenies (those that presented and maintained ChrE duplication in the previous study).

### 2.2 Propagation under no stress

The stability of the resistance in the 70 parental samples was analyzed after regrowing the samples in rich media lacking any antifungal stress. A smear of biomass of each investigated sample was taken from the glycerol stock and inoculated in 500 μl of YPD media. During 8 weeks, every 1–3 days, 50 μl of the sample was passed into a fresh 450 μl of the media. After 35 passages, single colonies were selected and stored in glycerol until further analysis.

### 2.3 Drug susceptibility test

Drug susceptibilities were obtained using Q-PHAST (Nunez-Rodriguez et al.,)^1^ where we placed plates with 96 spots on solid media plates on scanners and used a computational pipeline (https://github.com/Gabaldonlab/imageAnalysisPipeline_solid96wellPlates)) to measure and analyze the growth during the time. This python pipeline combines modified versions of colonyzer (Lawless et al., 2010) to measure the growth of the strains in plates, qfaR package (http://qfa.r-forge.r-project.org/) for calculating the fitness and, R scripts to plot and calculate different parameters. Note that the latest version of the pipeline, at the time of publication, is available at https://github.com/Gabaldonlab/Q-PHAST. Briefly, for each mutant, four single colonies were selected and grown overnight in 500 μl YPD medium at 37°C in a 96 deep well plate. The next day, the 3 μl of the saturated culture was diluted in 200 μl of sterile water and 5 μl was spotted on eight agar OmniTray plates. One of these plates contained control YPD medium, and the remaining seven plates contained YPD supplemented with 16 μg/ml, 8 μg/ml, 4 μg/ml, 1 μg/ml, 0.25 μg/ml, 0.0625 μg/ml, and 0.03125 μg/ml of anidulafungin or 256 μg/ml, 128 μg/ml, 64 μg/ml, 32 μg/ml, 16 μg/ml, 8 μg/ml, and 4 μg/ml of fluconazole. Plates were then incubated inside scanners placed in the incubators set up to 37°C, and the scanned images registered growth every 15 min during 24 h.

Fitness and susceptibility to antifungal drugs were calculated with a similar approach described before (Ksiezopolska et al., 2021) (**Supplementary Table S2**). Briefly, we used the area under the curve (nAUC), generated from the growth curves, of the samples grown at 64 μg/ml of flz and 0.25 μg/ml of ani. Additionally, we used the calculation of the area under the growth curve as the fitness estimates (AUC) for all the samples at all drug concentrations and YPD controls to get Minimum Inhibitory Concentration 50 (MIC_50_) and relative Area Under the Curve (rAUC). MIC_50_ values were assessed as the minimum concentration where the AUC relative to the no-drug control was below 50%. Whenever the sample did not present 50% of the inhibition at the highest used concentration, MIC_50_ was set as double of the maximum assayed concentration. The second used proxy, rAUC, was defined as area under the log2 drug concentration-vs-AUC, normalized by the maximum AUCMAX where there is no change in growth across all the tested concentrations. For a validation of the agar-based method described here by comparison with a liquid-based method, refer to Del Olmo et al. (2023) and Mixão et al. (2023). Outlier replicates that were wrongly detected as growing samples due to a technical issue, which is a reflected light of the corners of the agar plates, were eliminated from the analysis. Since FLZ parental samples were not resistant to ani, we tested their YPD-evolved progenies in all mentioned concentrations of ani only in the first replicates (FLZ_YPD_rep1); hence, MIC_50_ and rAUC were calculated only for these samples. FLZ_YPD_rep2 and rep3 were only grown on 0.25 μg/ml ani. Qualitative changes (maintained, decreased, or lost) of flz resistance were assessed manually by comparing rAUC, MIC_50_, and growth spots between YPD-evolved samples, parentals, and WT strains. We assessed the phenotype as the following: resistant for the ranges 0.95–1.6 for rAUC, ≥64 for MIC, >2.7 for nAUC, and when the growth spots were similar to those obtained for flz-resistant strains; decreased for 0.38–0.9 for rAUC, ≤64 for MIC, 1.26–9.22 for nAUC, and when growth spots were less intense; lost for 0.02–0.95 for rAUC, ≥64 for MIC, <3.1 for nAUC, and when there were no growth spots or they were less intense.

### 2.4 Spot tests

Samples were grown in 5 ml of YPD overnight. The cells were adjusted to an OD (optical density) of 1 and serially diluted 10 × 5 times. 5 μl of the final dilution was spotted on YPD agar plates containing 64 μg/ml fluconazole and on a control YPD-only plate. The growth was registered after 24 h of incubation.

### 2.5 Number of generations

The number of generations was estimated using the formula g = log_2_(N/N_0_), where g is the number of generations, N_0_ is the number of cells at the beginning of the incubation, and N is the number of cells at the end of the incubation, and mimicking the experimental setup of our *in vitro* evolution. Briefly, 10 strains (five parental strains: 2C_FLZ, 3H_FinA, 5F_ANI, 9F_AinF, and 10E_ANIFLZ and five evolved progenies: 2C_FLZ_YPD_1, 3H_FinA_YPD_2, 5F_ANI_YPD, 9F_AinF_YPD, and 10E_ANIFLZ_YPD), each in triplicates, were grown to saturation during 3 days. Each sample was then diluted 10×, and the number of cells was measured (N_0_). Since our experiment involved incubations of 24 h, 48 h, and 72 h between the passages, the samples were left to grow and the cells were measured accordingly (N). Next, the mean and standard deviations of the generation times were calculated for all the samples together for each incubation time and further multiplied by the number of passages of said incubations (11 for 24 h, 4 for 48 h, and 9 for 72 h). Presented is the sum of the number of generations considering also the standard deviations (+/−).

### 2.6 DNA extraction

A modified protocol from the MasterPure™ Yeast DNA Purification Kit was used to extract DNA. In brief, samples were grown overnight in liquid YPD at 37°C. Cells were pelleted and lysed with RNAse treatment at 65°C for 15 min. After 5 min of cooling down on ice, the samples were purified by the kit reagent by mixing, centrifugation, and removal of the debris, as described in the kit protocol. Furthermore, samples were left at −20°C with absolute ethanol for at least 2 h after which the DNA was precipitated for 30 min at 4°C. The pellet was washed in 70% ethanol and left to dry. TE buffer was used to resuspend the DNA. The Genomic DNA Clean & Concentrator kit (Zymo Research) was used for the final purification.

### 2.7 Whole-genome sequencing

Firstly, we sequenced genomes of 63 evolved samples: 60 AS samples (three replicates of 10 FLZ_YPD and three replicates of 10 FinA_YPD), and three whose parentals presented other chromosomal duplications (3H_AinF_YPD, 7B_AinF_YPD, 2G_ANIFLZ_YPD). The genomic DNA of these samples were sequenced in a total of 18 pools, and 17 of them were containing *N. glabratus* strains belonging to different phylogenetic clades (Carreté et al., 2018) (**Supplementary Table S3**). Secondly, in order to get an accurate genomic information of all samples presenting decrease in flz resistance, we sequenced 39 samples separately (**Supplementary Table S3**). In addition, note that all samples were pooled with DNA from divergent species and sequenced all together as described in Ksiezopolska et al. (2021), after confirming with Crossmapper (Hovhannisyan et al., 2020) the absence of read cross-mapping in the chosen sequencing design.

Genome sequences were obtained at the ultra-sequencing core facility of the CNAG. The short-insert paired-end libraries for the whole-genome sequencing were prepared with KAPA HyperPrep kit (Roche) with some modifications. In short, 1.0 μg of genomic DNA was sheared on a Covaris™ LE220-plus (Covaris). The fragmented DNA was further size-selected for the fragment size of 220 bp–550 bp with AMPure XP beads (Beckman Coulter). The size-selected genomic DNA fragments were end-repaired and adenylated, and Illumina platform-compatible adaptors with unique dual indexes and unique molecular identifiers (Integrated DNA Technologies) were ligated. The libraries were quality controlled on an Agilent 2100 Bioanalyzer with the DNA 7500 assay for size, and the concentration was estimated using quantitative PCR with the KAPA Library Quantification Kit Illumina Platforms (Roche). The libraries were sequenced on NovaSeq 6000 (Illumina) with a paired-end read length of 2 × 150 bp. Image analysis, base calling, and quality scoring of the run were processed using the manufacturer’s software Real Time Analysis (NovaSeq 6000 RTA 3.4.4).

### 2.8 Sequencing analysis

To find changing variants and aneuploidies, we analyzed the WGS data for all the samples generated here, sequenced after YPD evolution. In addition, we reanalyzed their FLZ/FinA/ANIFLZ-evolved parentals (resistant strains from Ksiezopolska et al., 2021) to identify changing variants. Furthermore, we reanalyzed the WT parental and the original YPD-evolved parental (strains that never acquired resistance) from Ksiezopolska et al. (2021) to remove background variation unrelated to the *in vitro* evolution experiment. For all the samples where we did pools of different species, we depooled them as in Ksiezopolska et al. (2021).

In order to evaluate the presence of aneuploidies and changing small variants in these samples, we used perSVade (Schikora-Tamarit and Gabaldón, 2022) for quality control, read mapping, and variant calling. To get high-quality reads, we used the “trim_reads_and_QC” module, which uses Trimmomatic (Bolger et al., 2014) to trim the reads and fastqc (Babraham Bioinformatics - FastQC A Quality Control tool for High Throughput Sequence Data, 2023) for quality control. In addition, we used multiqc (Ewels et al., 2016) to perform an integrated quality control. We then mapped these trimmed reads with the “align_reads” module, which uses bwa mem (v0.7.17, http://bio-bwa.sourceforge.net/bwa.shtml). We used the v_s02-m07-r35 *N. glabratus* reference genome from Candida Genome Database (Skrzypek et al., 2017). To validate our datasets, we used the “get_cov_genes” module of perSVade (using mosdepth (Pedersen and Quinlan, 2018)) to calculate the coverage per gene, which showed that all our datasets have a median coverage > 100×. Note that the results of “get_cov_genes” were also useful to call aneuploidies and copy-number variants (CNVs) in the pure samples (those that included a single *N. glabratus* strain).

To call small variants (SNPs and small IN/DELs), we used the “call_small_variants” module of perSVade, with a different strategy for each sample type. First, for pure samples (each with a single strain), we used the arguments “-p 1 –callers HaplotypeCaller,bcftools,freebayes –min_AF 0.9 -c 15”. With this, the module uses GATK HaplotypeCaller (Poplin et al., 2017), bcftools (Li, 2011), and freebayes, with custom filters as in Ksiezopolska et al. (2021), ploidy 1 configuration, and keeping only positions with a coverage >15×. Second, for pooled samples (with several strains from different clades), we used the arguments “–callers freebayes –min_AF 0 -c 2 –pooled_sequencing”. With this, the module uses the “pool” mode from *freebayes* (v1.3.1 https://arxiv.org/abs/1207.3907), keeping only positions with a coverage >2×. To annotate these variants, we used the “annotate_small_vars” module of perSVade, which uses VEP (McLaren et al., 2016) to annotate the functional effect of each variant. For this functional annotation, we used the gff file corresponding to the reference genome from CGD.

To find aneuploidies in each strain within pooled samples, we measured the coverage of each strain from its unique genomic features (i.e., SNPs found only in that sample) as compared with the other members of the pool. In order to achieve this, we first defined as “private SNPs” of a strain those that were not expected in any of the other samples of the pool. We defined as “expected SNPs” those that were called in the parental WT strains (not subjected to any *in vitro* evolution). We found that most strains (all non-CBS138 strains) had at least 14,946 positions with such private SNPs. The read depth of each of these SNPs was taken as a proxy for the coverage of the corresponding sample. However, all CBS138 strains had less than three of these, suggesting that considering private SNPs is not enough to resolve the coverage of all strains. To overcome this, we identified “private no SNPs” in a sample as positions without SNPs where all the other members of the pool had some SNP. We calculated the coverage of each “no SNP” as the “total coverage in a position”—”sum of the coverage of each SNPs in the other samples”. This yielded >16,655 “no SNPs” for all CBS138 samples, suggesting that considering “no SNPs” could be useful. In order to avoid errors derived from inaccurate variant calling, we considered as “private SNPs” those that were “high-confidence” in a given strain (called by three algorithms in the parental and with at least 90% of reads of the position supporting that SNP, as in Ksiezopolska et al. (2021), and not called in any of the other members of the pool. Importantly, we validated that most of these “expected” SNPs were also called in the pools (>98.47% in all samples). Similarly, we only considered “private no SNPs” as those positions where the strain had no called SNPs and all the other members had “high-confidence” SNPs. We found that most positions that were expected to include some “no SNP” yielded the expected SNPs in the pooled sequencing (>99.04% in all pools). Taken together, these observations indicate that the combination of “private SNPs” and “private no SNPs” can be useful to measure the read depth of each strain from the pooled sequencing. We thus obtained the coverage of each position as the reads covering the “private SNPs” (or “no SNPs”) of a given sample. We also calculated a “relative coverage” measure by normalizing the coverage of each position by the median coverage across all positions of chromosomes not expected to have aneuploidies in any strain (chromosomes B, C, D, F, G, H, J, K, M). This “relative coverage” was expected to be proportional to the copy number in a given position, and we used it to identify aneuploidies in pooled strains.

To detect aneuploidies in strains within pure samples (with only one strain), we used the per-gene coverage results from perSVade (see above). We calculated a “relative coverage” measure by normalizing the coverage of each gene by the median coverage across all genes of chromosomes not expected to have aneuploidies in any strain (chromosomes B, C, D, F, G, H, J, K, M).

To identify small variants lost during the YPD evolution in pooled samples, we checked whether the expected resistance-conferring mutations of each strain were identified with the pooled calling strategy. We only considered the expected variants around the important genes (*FKS2*, *PDR1*, *FKS1*, *ERG11*, *ERG3*, *CDR1*, *CNE1*, and *ERG4*) as defined in Ksiezopolska et al. (2021).

To identify small variants that were lost or acquired during the YPD evolution in pure samples, we analyzed the variants of 39 strains sequenced in pure samples and their parentals (resistant strains and background parentals). Note that 24 of these samples are all those samples that show a decrease in resistance to flz. We defined as “high-confidence” small variants those that passed the filters of the three algorithms with at least 90% of reads of the position supporting that SNP. In addition, we defined as “low-confidence” variants those that were called by any algorithm. For each strain after YPD evolution, we defined “background variants” (all “low-confidence” variant found in any of the WT/YPD pre-*in vitro* evolution strains) and “resistance variants” (“high-confidence” variants in the resistance parental but absent in the “background variants”). We defined as “new variants” those that were “high confidence” in the after-YPD strain and absent in both the “background variants” and in the “low confidence” variants from the resistance parental. We define as “lost variants” those “resistance variants” that were absent in the “low confidence” variants of the after-YPD strain.

To identify CNVs (deletions and duplications) that were lost or acquired during the YPD evolution in pure samples, we analyzed the per-gene coverage of 39 strains sequenced in pure samples and their parentals (resistant strains and background parentals). To correct for intrinsic coverage biases, we defined the log2cov_vsYPD for each gene as the log2 ratio between the relative coverage and the relative coverage in the background YPD parental strain (from Ksiezopolska et al., 2021). For each strain, we defined genes with “high-confidence” duplications (with log2cov_vsYPD >1 and a relative coverage >1.8) and/or “low-confidence” duplications (with log2cov_vsYPD >0.5 or a relative coverage >1.3) In addition, we defined genes with “high-confidence” deletions (with <50% of the gene covered) and/or “low-confidence” deletions (with <50% of the gene covered or a relative coverage <0.1). We then defined “background CNVs” (deletions or duplications present in all background WT/YPD parentals with “low confidence”) and “resistance CNVs” (“high-confidence” CNVs in the resistance sample absent in the “background CNVs”). Finally, we defined as “new CNVs” those “high-confidence” CNVs from the after-YPD strain that were absent in the “background CNVs” and in the “low-confidence” CNVs from the resistant sample. In addition, we define as “lost CNVs” those “resistance CNVs” that were absent in the “low confidence” CNVs of the after-YPD sample. Note that for duplications, we only considered genes within chromosomes that had no aneuploidies in any strain (chromosomes B, C, D, F, G, H, J, K, M).

Finally, we filtered out variants that appeared to be new (or lost) in multiple strains, as these are likely mapping or variant calling artifacts.

### 2.9 PCR and Sanger sequencing

The loss of *PDR1* mutations and *FKS1* after the *in vitro* evolution was confirmed by PCR and Sanger sequencing. The PCR primers for *PDR1* are FWD—TCAAAATGCACCCAGTTCGA and REV—TCTAACGGGTTGGCAATCGA, and those for *FKS1* are FWD—TGGTCACCCGGATTTCATCA and REV—TCACCCATACCAGCACCAAT. PCRs were carried out by using Taq DNA polymerase from DongShengBio. The reaction mixture included primers of concentration of 0.4 μM, 20 μl Taq DNA polymerase, 1 μl liquid sample grown for 24 h in YPD, and water up to a final volume of 40 µl. Optimase ProtocolWriter™ was used to develop conditions.

## 3 Results

### 3.1 Using *in vitro* evolution coupled with phenotyping to assess long-term stability of drug resistance

**Figure 1:**
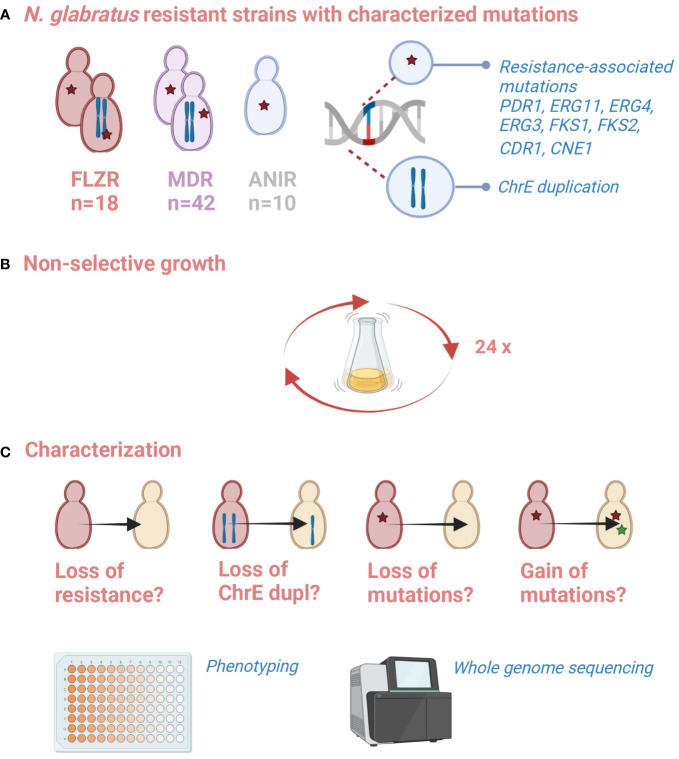
Schematic representation of the experiment. **(A)** A battery of resistant strains with characterized resistance-associated mutation is available from a previous study (Ksiezopolska et al., 2021). **(B)** These strains were propagated in non-selective conditions for 2 months (35 passages). **(C)** Changes in susceptibility phenotypes, and the presence of previously or newly acquired genetic alterations was assessed. Created with biorender.com.

We used an *in vitro* evolution approach to test the perdurability of acquired resistance in a set of 70 mono- and multidrug-resistant *N. glabratus* strains (**Supplementary Table S1**) obtained in a previous study (Ksiezopolska et al., 2021). This set included 18 flz-resistant, 10 ani-resistant, and 42 flz and ani-resistant (MDR) samples obtained from directed evolution experiments. Mutations present in these strains that were acquired under drug exposure have been characterized previously through whole-genome sequencing and potentially represent diverse genetic mechanisms of resistance, including mutations in *FKS* genes in ani-resistant strains as well as mutations in *PDR1*, *ERG11*, or ChrE aneuploidies in flz-resistant strains (Ksiezopolska et al., 2021). In addition, other genes were associated with the resistance phenotype as they accumulated mutations during drug adaptation in independent populations, including *ERG3* (confirmed as resistance driver in that study), *ERG4*, *CDR1*, or *CNE1.* To assess the stability of the resistance phenotype and resistance-associated mutations after prolonged growth in the absence of drug exposure, we serially passaged the strains in a rich medium (YPD) free of antifungal agents during 8 weeks, which we estimated to represent 140 ± 20 generations (see **Figure 1** and Materials and Methods).

We placed a particular focus on 20 strains carrying ChrE duplications (10 flz-evolved samples and their 10 progenies further evolved in ani, which showed persistent chromosomal alteration mentioned in the introduction), which will be further referred to as aneuploid samples (AS), and for which we carried out the experiment in triplicates. In total, 110 YPD-evolved strains resulting from 70 resistant parental strains were analyzed (50 non-aneuploid samples plus triplicates of 20 AS samples).

**Figure 2:**
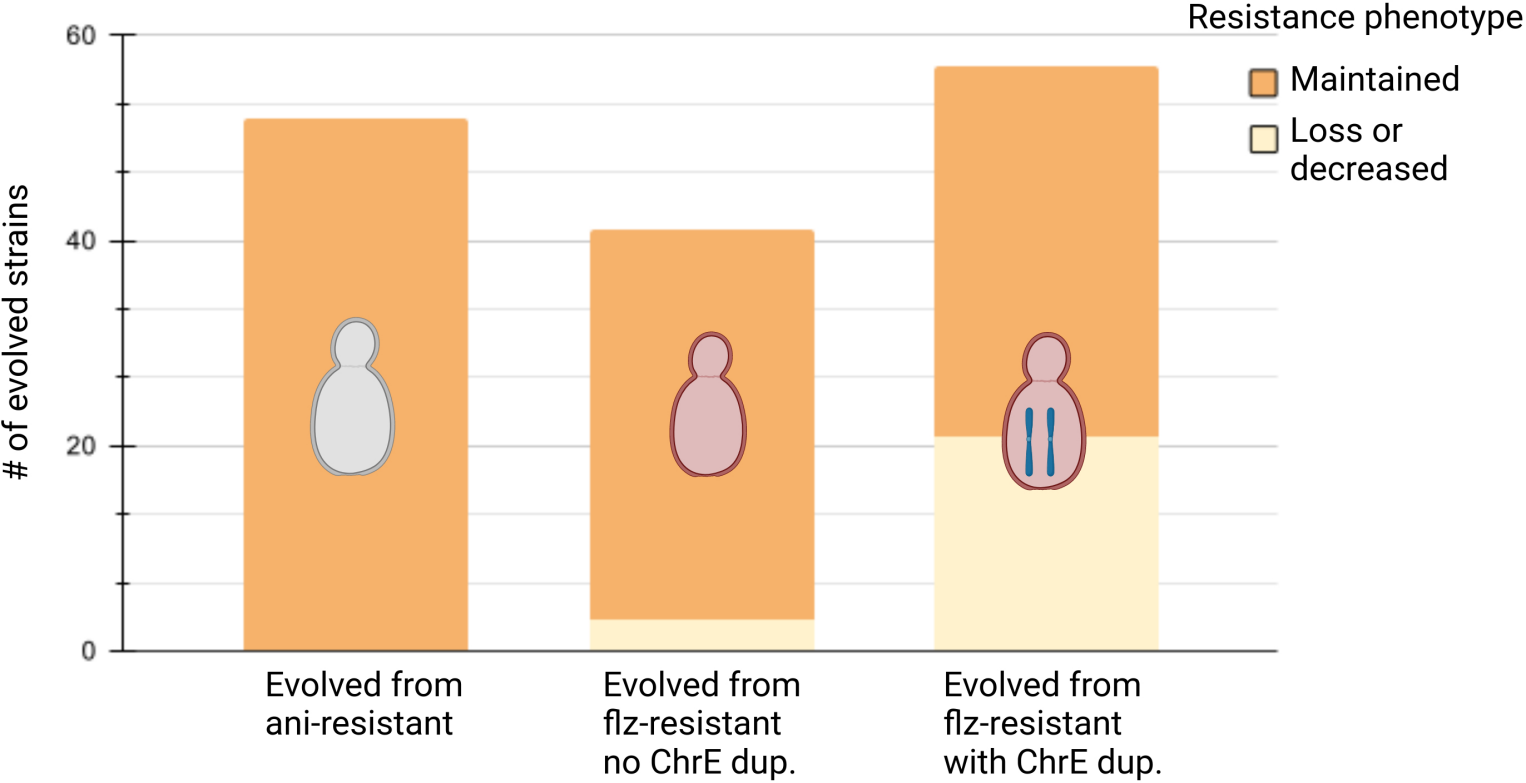
Summary of phenotypic evolution. Summary of phenotypic evolution of resistant strains after propagation in non-selective conditions for ani-resistant strains, flz-resistant strains without ChrE duplication, and flz-resistant strains with ChrE duplication, in this order. Maintenance or resistance correspond to the corresponding drug (ani for ani-resistant and flz for flz-resistant). Created with biorender.com.

**Figure 3:**
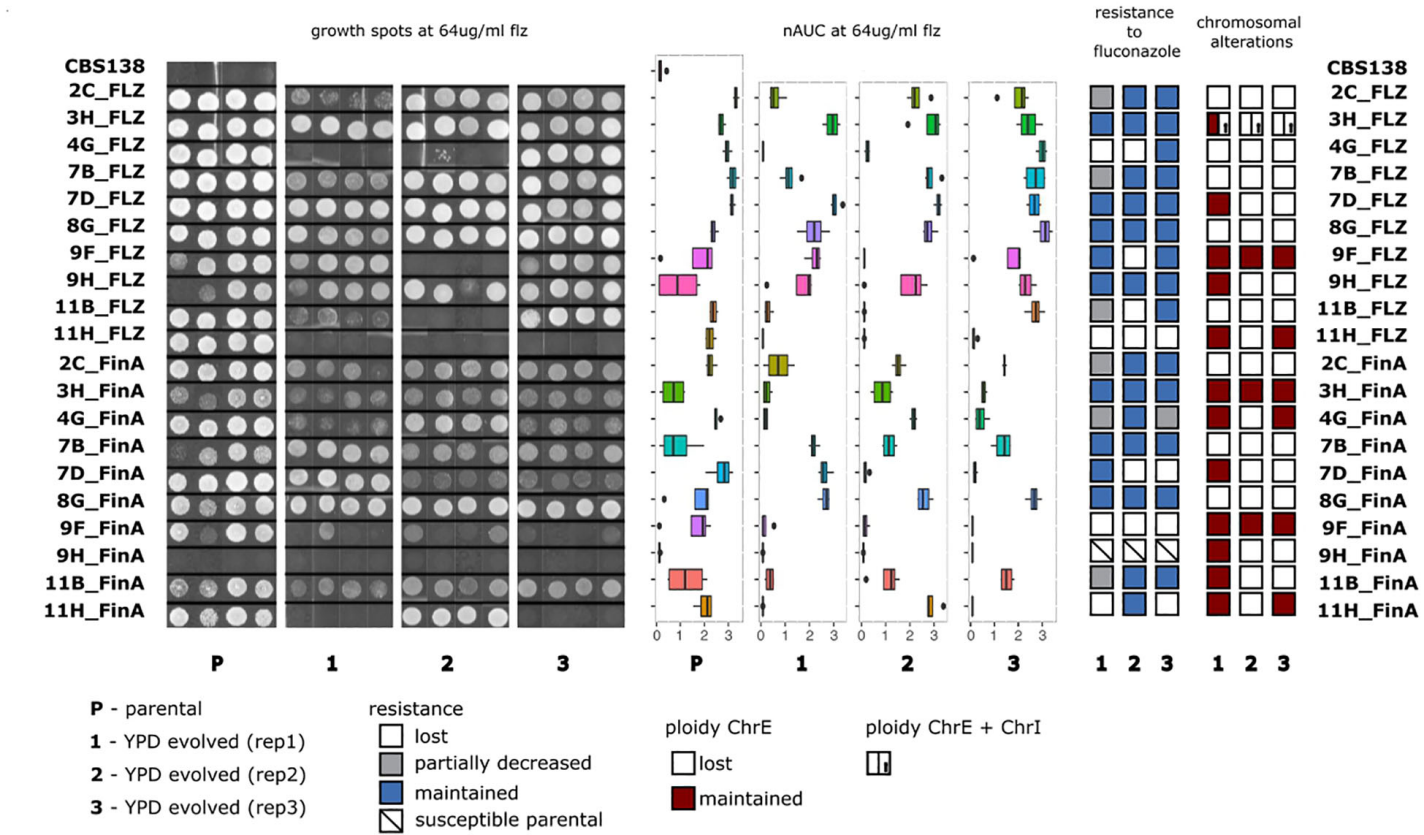
Analysis of changes in flz resistance and aneuploidies in AS samples. From left to right: Growth spot assays, areas under the growth curves (nAUC) of parentals and evolved replicates of the AS samples grown at 64 μg/ml of flz; stability of the resistance phenotype and the chromosomal duplications in YPD-evolved replicates.

We evaluated the susceptibility of the evolved samples by spot test and image-based quantification of colony growth (Materials and Methods). For this, we first used agar plates supplemented with seven increasing concentrations of the drugs and a no-drug control plate, spotted them with the samples, and registered the growth with scanners. Image analysis was used to calculate growth curves, minimal inhibitory concentrations (MIC_50_, drug concentration at which 50% inhibition of the growth is observed as compared with growth on the plate without the drug), areas under the growth curve (nAUC) for different concentrations, and the relative area under the curve (rAUC). rAUC has been previously proposed as a quantitative proxy for susceptibility that is more robust than MIC_50_ (see Ksiezopolska et al. (2021) for a detailed explanation) and is defined as the area under the drug concentration-versus-relative fitness curve (AUC), normalized by the maximum AUC_MAX_ where there is no change in fitness across the entire range of concentrations (**Supplementary Figure S1**). Additionally, we visually examined growth spots. Finally, these different susceptibility measures (MIC_50_, rAUC, nAUC, visual assessment of spot growth) for the parental (resistant) and evolved strains were compared which provide a qualitative assessment of “maintenance,” “loss,” or “decrease” of the resistance phenotype (Materials and Methods). The overall results are shown in **Figures 2**, **3** and in **Supplementary Table S2**.

### 3.2 Acquired drug resistance is long-lived in non-selective conditions

Strikingly, our results show that all 52 ani-resistant samples retained the ani resistance phenotype after evolution in YPD (**Figure 2**, **Supplementary Figures S2–S4**). Interestingly, the evolution of flz resistance in YPD was markedly different depending on the presence of ChrE aneuploidies. Susceptibility to flz in non-AS increased in only three YPD-evolved samples (3/41, 7%) (**Figure 2** and **Supplementary Figures S4, S5**), as compared with 36.8% (21/57) in the AS set (**Figure 3**, **Supplementary Figure S3**). This almost five fold difference in the propensity to diminish resistance indicates a higher plasticity of the aneuploidy-aided resistance as compared with resistance based on point mutations. Interestingly, one-third of the AS samples that decreased in flz susceptibility (7/21) retained intermediate levels of susceptibility, still higher than their original native WT drug-susceptible ancestors.

### 3.3 Loss of chromosome E duplicates is common but largely uncoupled to loss of flz resistance

**Figure 4:**
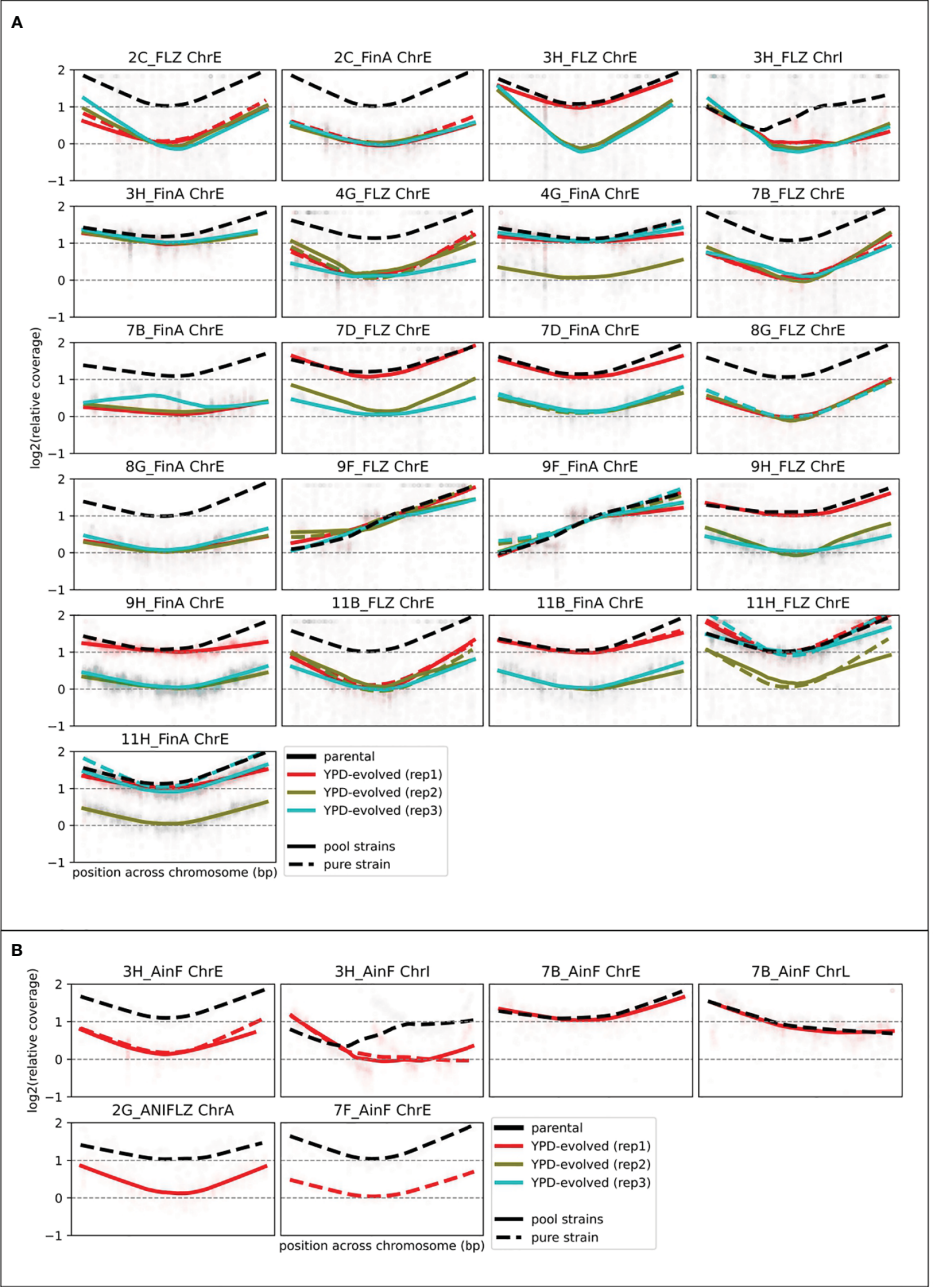
Aneuploidy assessment from depth of coverage of strain-specific SNP coverage. Relative coverage (as compared with the median of non-aneuploid chromosomes) plots for several genomic positions before (black) and after (colored) 8 weeks of growth in YPD. We log_2_-normalize the data by the coverage in the YPD-evolved sample (of the corresponding strain (see Ksiezopolska et al. (2021)) to correct strain-specific biases. Coverage of YPD-evolved lines (colored) was measured from the sequencing of pooled samples (see Materials and Methods). This approach only allowed the calculation for some positions, which explains why there are gaps in these figures. Each panel corresponds to one aneuploid chromosome in a strain. **(A)** Shows the AS samples (10 FLZ samples with their 10 FinA progenies (parentals of this study) with three YPD-evolved replicates. **(B)** Shows the four additional samples for which we investigated chromosomal alterations that also included changes in other chromosomes.

To gain a mechanistic understanding on the causes of resistance loss and to assess the overall stability or previously acquired resistance-conferring mutations, we selected samples for individual or pooled whole-genome sequencing. To increase the number of analyzed strains with the available budget, we used a pool-sequencing strategy in which DNA from several *N. glabratus* strains belonging to genetically different clades (therefore with identifiable SNP patterns) were pooled in equal proportion and a single sequencing library was prepared for each pool (**Supplementary Table S3**). The analysis of the relative coverage of strain-specific alleles in the different chromosomes enabled us to identify the aneuploidies present in each pooled strain (see Materials and Methods). We could calculate the coverage for at least 882 genomic positions with clade-specific variants in all aneuploid chromosomes. We confirmed the results in 39 samples that were also sequenced separately and concluded that our strategy resolves the presence of aneuploidies in an accurate and cost-effective manner. The presence of aneuploidies was investigated in such a way in all 60 YPD-evolved AS samples and in four additional samples, including three samples whose parentals presented other types of aneuploidies. The final set included 55 samples that presented alterations only in ChrE, four in ChrE and ChrI, one in ChrE and ChrL and one in ChrA (**Figure 4**).

Our results (**Table 1**) revealed diverse combinations of chromosome loss and decrease of resistance that suggest a non-deterministic relationship between the two events. The duplicated ChrE was lost in 41 (41/63, 65%) of the investigated strains, but only in 12 (12/41, 29%) of these the aneuploidy loss was accompanied by a decrease in resistance to flz. In addition, this decrease was intermediate for four of these strains. Thus, in the majority of the samples that lost the duplicated ChrE, this loss was not accompanied by a corresponding loss of flz resistance, suggesting the retention of the other (more likely) genetic drivers (identified in (Ksiezopolska et al., 2021)). Conversely, we also observed the maintenance of the ChrE duplication in 11 strains that nevertheless showed a decrease in resistance, suggesting that other genetic alterations may have driven the increase of susceptibility. The single strain harboring ChrL aneuploidy maintained it, whereas aneuploidies affecting ChrI and ChrA were always lost. Loss of ChrI aneuploidy only resulted in increased susceptibility in a single strain where ChrE was also lost. Overall, loss of ChrE was more associated with the maintenance, rather than the loss, of the resistance phenotype, and there was no statistically significant association between ChrE loss and loss of resistance (p-value 0.163, Fisher exact test). Altogether, our results suggest that ChrE duplications tend to be lost during evolution in YPD, but that this only sometimes leads to increased flz susceptibility. Hence, other mutations present in the resistant parental strains that initiated the experiments are likely playing a more important role in the drug resistance phenotype, as compared with ChrE duplications.

**Table 1:** Analysis of changes in flz resistance vs. chromosomal alterations.

		ChrE duplication		ChrI duplication		ChrL duplication		ChrA duplication	
flz resistance		Lost	Maintained	Lost	Maintained	Lost	Maintained	Lost	Maintained
	Decreased	4	3	1	0	0	0	0	0
	Lost	8	8	0	0	0	0	0	0
	Maintained	27	10	3	0	0	1	1	0
		39/60	21/60	4/4	0/4	0/1	1/1	1/1	0/1
		65%	35%	100%	0%	0%	100%	100%	0%

Note that 9H_FinA was excluded as it was susceptible to flz at the beginning of the experiment.

### 3.4 High stability of resistance-associated mutations

Given the potentially higher phenotypic relevance of point mutations noted above, we mined our sequencing data for the presence of resistance-related point mutations in *FKS1*, *FKS2*, *ERG11*, *ERG3*, *ERG4*, *PDR1*, *CDR1*, and *CNE1* genes (see Materials and Methods). Given the recurrent presence of non-synonymous mutations appearing during drug exposure, these seven genes have been previously considered relevant for drug adaptation (Ksiezopolska et al., 2021), and we consider them here as resistance-associated without necessarily implying that they are causative of the resistance. All examined samples, except three (59/62, 95%), retained the relevant mutations acquired during drug adaptation, indicating a much higher stability of point mutations as compared with aneuploidies. Unexpectedly, all three samples that lost the resistance-associated point mutations are progenies of the same WT strain (BG2). One lost mutation involved a missense mutation (L280F) in *PDR1*, another one lost an insertion (V339/VE) in *PDR1*, and the remaining one lost a STOP codon (Q1230*) in *FKS1.* In all these cases, the mutated positions reverted to the wild-type configuration. The two samples where mutations in *PDR1* gene were lost showed a decrease in flz resistance, despite the retention of a ChrE aneuploidy in one of them, suggesting a larger effect of the *PDR1* mutation on the phenotype. Targeted sequencing surrounding the mutated positions in *PDR1* in all 12 BG2 progenies confirmed the whole-genome sequencing results and showed no other changes in the investigated regions in any of the tested samples. We investigated the loss of the STOP codon (Q1230*) in more detail by visually inspecting read alignments. In the sample mentioned above, TAA (STOP) reversed to CAA (Gln), and we found that another YPD-evolved replicate of the same parental sample had a different mutation in the same codon, TAA changed to TCA (Ser), also resulting in the loss of the premature stop codon. None of these mutations impacted the susceptibility to ani, likely because all strains retained *FKS2* missense mutations, which were present in the parental strains in addition to the *FKS1* truncation. The fact that the four cases that lost previously acquired genetic alterations are descendants of BG2 may indicate that gain of function mutations in *PDR1* or truncating mutations in *FKS1* have a fitness cost in the tested growth conditions and, specifically, in the BG2 genetic background, irrespective of their role in flz or ani resistance.

To increase our genotyping resolution and gain further insights on possible genetic mechanisms driving the increase in flz susceptibility, we individually sequenced the whole genome of all 24 samples presenting changes in the resistance phenotype (**Supplementary Table S4**). Four of the investigated samples (4/24) lost previously acquired nonsynonymous mutations: one lost changes in *PDR1*, *PEX17*, and *CAGL0E06182g*, one in *PDR1* only, one in *FKS1*, and one in *RPD3*. Losses in *PDR1* and *FKS1* were confirmed by Sanger sequencing as mentioned before. Importantly, we also identified newly appearing mutations in the resistance-associated genes, which were more common. We observed that 13/24 samples presented new protein-altering mutations in *PDR1* and 2/24 in *CDR1* which we suspect to have the largest impact on the loss of resistance. The acquisition of these mutations happened regardless of the loss or maintenance of ChrE duplication: six samples that lost the duplication presented new *PDR1* mutations, and nine that maintained it showed new mutations in *PDR1* (seven samples) or *CDR1* (two samples). Hence, these protein alterations are likely to explain the loss of resistance in these 15 samples.

For the nine remaining samples that lost flz resistance, the relationship between the observed mutations and the increase of susceptibility is less obvious. Two samples (both from EF1620 WT background) did not present any new protein-altering mutation, but they had lost ChrE duplication, pointing to a possible role of the aneuploidy in this phenotype. Four other strains that lost ChrE duplications presented other mutations, and therefore, there is not a direct link between the mutation and the loss of resistance: one had a new protein-altering mutation in *EPA7*, encoding an adhesin; one acquired a new mutation in the ortholog of *S. cerevisiae LAM6*, involved in intracellular sterol transfer, and lost the previously acquired one in *RPD3* (discussed above); one had a new mutation in the ortholog of *S. cerevisiae LRE1*, involved in control of cell wall structure and stress response; and the other one had no protein-altering mutation but suffered a concomitant loss of ChrI. Two other samples that retained the ChrE duplication but lost the resistance may point to new mechanisms involved in the phenotype loss: one presented new protein-altering mutations in the ortholog of *S. cerevisiae FMO1*, encoding a monooxigenase involved in protein folding and ER localization, and *TBF1*, a telomere repeat binding factor; the remaining one did not present any new protein-coding alteration, suggesting that non-coding mutations may be involved. Finally, one sample whose parental did not have any chromosomal alterations acquired new mutations in *GAL11A* (Q477H and Q478*), a gene encoding a protein with a critical role in regulation of multidrug resistance in *N. glabratus* (Thakur et al., 2008) which we suspect to impact on the decrease in susceptibility to flz. Lastly, we also individually sequenced the whole genome of 16 YPD-evolved samples that maintained susceptibility to flz. We observe that only one (1/16) of these samples presented a new variation in the (S447W) *PDR1* gene.

## 4 Discussion

Resistance to antimicrobials is growing due to the ability of microbes to adapt to drugs through the acquisition of genetic alterations. However, how long-lived these alterations and the resulting resistant phenotypes are is still poorly understood. This study aimed to assess the stability of secondarily acquired resistance phenotypes and their genetic drivers in *N. glabratus* after cultivation under non-selective growth conditions. For this, we used a unique combination of *in vitro* evolution, high-throughput phenotyping, and whole-genome sequencing and took advantage of the availability of a well-characterized collection of 70 mono- and multidrug-resistant strains previously obtained by directed evolution (Ksiezopolska et al., 2021).

Overall, we observed a high stability of the resistance phenotype, particularly for ani, which was retained in all samples, as opposed to 83% for flz. A higher stability of the ani resistance phenotype is consistent with our earlier observation that ani-resistant isolates showed similar fitness values in antifungal-free conditions as their wild-type parentals, whereas growth on YPD of flz-resistant isolates was more impaired (Ksiezopolska et al., 2021). This may imply a higher fitness cost (in optimal growth conditions) of flz resistance as compared with ani resistance, resulting in a higher propensity to be lost. In contrast, in that previous study (Ksiezopolska et al., 2021), we observed that, when ani-resistant strains were exposed to flz, ani resistance was lost in 8.4% of all tested samples, whereas flz resistance was more likely to be retained in cells exposed to ani (lost in 2.1% of the tested samples). This trend is opposite to what we observed here in optimal growth conditions. On a similar note, the samples that lost resistance to the first drug when treated with a second one in our previous study all maintained the resistance phenotype when evolved in YPD in this study. Finally, our previous study identified flz cross-resistance in ani-evolved samples carrying *ERG3* mutations. In this study, all these samples maintained this cross-resistance after evolution in YPD. All these observations highlight that the propensity for maintaining or losing the resistance phenotype is dependent on the environmental conditions, likely due to context-dependent fitness cost of the underlying mutations. Intriguingly, these results also suggest that the fitness cost of flz-resistance associated mutations may be lower under exposure to ani, which reinforces the cross-talk between ani and flz resistance. Further studies may explore how such processes contribute to the relatively high rate of multidrug resistance observed in *N. glabratus* (Arendrup and Patterson, 2017).

Aneuploidies are often observed after drug exposure (Marichal et al., 1997; Perepnikhatka et al., 1999; Yang et al., 2019; Ksiezopolska et al., 2021) and are generally considered a transient mechanism to cope with drug stress. We observed that chromosomal aneuploidies were often lost in optimal conditions, reinforcing their suggested temporary role in adaptation to drug resistance (Rustchenko, 2007). Nevertheless 22 samples (35%) retained ChrE aneuploidy after 2 months of growth in non-selective conditions, which is not negligible and would suggest that aneuploidy-aided flz resistance could persist for long periods of time in *N. glabratus* populations in untreated patients or in the environment. Again, this observation made in optimal growth conditions contrasts with observations of nearly 100% retention under ani exposure for a similar period of time (Ksiezopolska et al., 2021). This suggests a higher stability of aneuploidies under a stress condition imposed by ani treatment, even though this stress is different from the one that originated the aneuploidy. In clinical settings, this would mean that the acquired duplications could be also stable after the change of drug regime. Importantly, however, in our experiment ChrE loss and loss of flz resistance were largely uncoupled. This may be explained because all of our aneuploid parental strains bear also *PDR1* sequence alterations, which were often retained. This suggests a transient and less determinant role of ChrE duplications in flz resistance, at least after *PDR1* mutations have appeared, and highlights the importance of *PDR1* mutations in driving flz drug resistance. Additionally, we observed that 62.5% of the YPD-evolved samples that decreased in resistance to flz presented reversions or new protein-altering mutations in *PDR1* or *CDR1* genes, strongly suggesting these were responsible for the phenotypic change. Overall, our results support a more determinant role of *PDR1* point mutations in stable flz resistance phenotype, and a less determinant role of the commonly observed ChrE aneuploidies, which likely play a transient role and allow a quick, initial but incomplete adaptation to the stress exerted by the drug. Other mutations associated with the loss of resistance may hint to novel mechanisms related to drug-adaptation and the resulting fitness costs. Although a direct association is difficult with our data and requires further research, some working hypotheses can be contemplated. These include the potential involvement of *GAL11A*, a gene involved in transcriptional regulation and which has been already related to drug resistance (Thakur et al., 2008), the orthologs of *S. cerevisiae LAM6*, involved in intracellular sterol transfer*, LRE1*, involved in control of cell wall structure and stress response, and *FMO1*, with roles in protein folding and ER localization.

Importantly, our findings of a significant number of new mutations appearing in key resistance genes such as *PDR1*, *CDR1*, and *FKS1* during the evolution under non-selective conditions suggests a fitness cost of the previously acquired resistance-conferring mutations in these genes. Importantly, this cost would not always be linked to the resistance phenotype, as most of the newly acquired mutations did not lead to a loss in this phenotype. Finally, our experiment included strains from broadly different genetic backgrounds—e.g., different *N. glabratus* clades sensu (Carreté et al., 2018)—and we observed some trends in this regard, such as a tendency to revert or compensate for mutations in *PDR1* and *FKS1* in the *BG2* background. Overall, our observations underscore the complex relationships between phenotype, genotype, and environment.

Altogether, our results indicate a relatively long-lasting stability of acquired resistance in the absence of selective conditions. Of note, our study is limited to *in vitro* conditions and therefore cannot consider external factors that may influence fitness of resistant strains, including activity of the host immune system, or exposure to clinical settings (e.g., use of disinfectants). Nevertheless, our results are consistent with previous, smaller-scale studies (Borst et al., 2005; Imbert et al., 2016; Hatwig et al., 2019) and extend these by providing a more comprehensive, quantitative, and mechanistic understanding of the process based on a large number of distinct samples in strictly controlled conditions. The implications of these observations are of major clinical relevance. For instance, high stability of the resistance phenotype could explain the expansion of resistant clones through non-exposed environments such as untreated patients, doctors, hospital devices, or non-clinical environments. Understanding factors that promote retention or loss of resistance phenotypes will be important for the design of efficient therapies and the implementation of measures to contain outbreaks caused by resistant strains. Future studies should ideally consider alternative clinically relevant conditions and explore other related phenotypes such as tolerance.

## Data availability statement

The datasets presented in this study can be found in online repositories. The names of the repository/repositories and accession number(s) can be found below: https://www.ncbi.nlm.nih.gov/bioproject/PRJNA1064689.

## Author contributions

EK: Data curation, Formal analysis, Investigation, Methodology, Validation, Visualization, Writing – original draft, Writing – review & editing. MS-T: Data curation, Formal analysis, Investigation, Methodology, Software, Validation, Visualization, Writing – review & editing. JC-R: Investigation, Methodology, Visualization, Writing – review & editing. TG: Conceptualization, Formal analysis, Funding acquisition, Investigation, Project administration, Resources, Supervision, Visualization, Writing – original draft, Writing – review & editing.

## Footnotes

1 Nunez-Rodriguez JC, Schikora-Tamarit MÀ, Ksiezopolska E, and Gabaldón T. Q-PHAST: simple, large-scale quantitative phenotyping and antimicrobial susceptibility testing. (in preparation).
