# Supplementary material for: Long-term stability of acquired drug resistance and resistance associated mutations in the fungal pathogen Nakaseomyces glabratus (Candida glabrata)
Source: Front Cell Infect Microbiol. 2024 Jul 15;14:1416509. doi: 10.3389/fcimb.2024.1416509 (PMC11284152; doi:10.3389/fcimb.2024.1416509)
Supplement: Supplementary file 5 [file Presentation_1.pdf]

Supplementary Material

Long-term stability of acquired drug resistance and resistance associated mutations in the fungal pathogen Nakaseomyces glabratus (Candida glabrata)

Ewa Ksiezopolska[1,2], Miquel Àngel Schikora-Tamarit[1,2], Juan Carlos Nunez-Rodriguez[1,2] and Toni Gabaldón[1,2,3,4*]

*Correspondence:
Corresponding Author
toni.gabaldon@bsc.es

1 Supplementary Table Legends

Supplementary Table S1. Strains used in this study. Table includes mutant names, antifungal treatment of the experimental evolution, clade, wild type susceptible parental strain, experimental evolution replicate, chromosomes that were altered and SNPs in the main genes. Note that numbers in the Replicate correspond to a WT strain.

Supplementary Table S2. Stability of the resistance and chromosomal duplications. Table presents information about chromosomal alterations presented in the parental strain, loss and maintenance of them in YPD evolved mutants, r (growth rate) and Area Under the Curve (nAUC) at 64ug/ml flz and 0.25ug/ml ani combined with MIC_{50} and rAUC, all with standard deviations of the technical replicates. Wild type strains are marked in gray and resistant parental strains are marked in bold. Additionally, in the tab for flz susceptibilities there is information on parentals chromosomal alterations and chromosomal alterations after YPD evolution.

Supplementary Table S3. Sequencing pools. Information about the sequencing pools and samples sequenced in this study. First part of the table presents the pools of *Nakaseomyces glabratus (Candida glabrata)* strains coming from different clades (except pool 16 which was sequenced without other *N. glabratus* stains) while the second part of the table presents 39 samples that were individually sequenced.

Supplementary Table S4. Genomic changes in 39 individually sequenced samples. The table is divided into two parts, first shows the changes in 24 samples that presented an increase in the flz susceptibility and the second part shows additional 15 samples that did not present changes in flz susceptibility. The columns are: sample number, sampleID, type of the mutation (duplications, deletions or small_vars (small variation), is_protein_altering (TRUE or FALSE if the mutation is affect the protein), Gene (ID of the gene), final_name (name if applied), type_var ('resistance_var_kept' (a variant that appeared in the original drug evolution and was kept in the subsequent YPD evolution), 'new_var' (a variant that appeared newly in the YPD evolution) or 'resistance_var_lost' (a variant that appeared in the original drug evolution and

was lost in the subsequent YPD evolution), #Uploaded_variation (an identifier of the variant: DUP for duplication, DEL for deletion, mis for missense mutation, FS for frameshift, PTC for premature termination codon), short_variant_effect (indicates the impact of the variant on the gene), description of the gene and comment on possible cause of the decrease in flz resistance.

2 Supplementary Figures

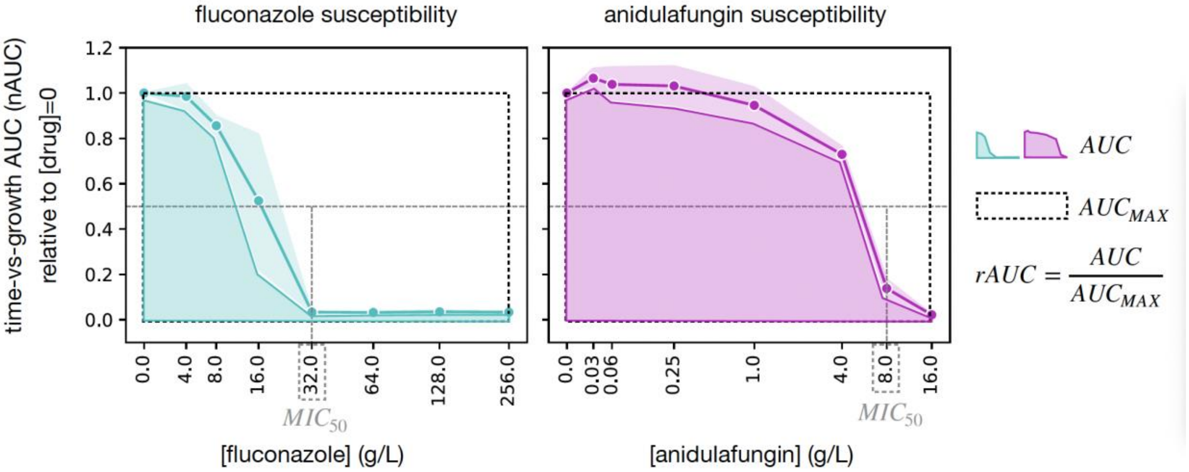

Supplementary Figure S1. Drug susceptibility measurements used here. Relative fitness (the ratio between fitness in each drug concentration versus the no-drug condition) at various fluconazole (left) and anidulafungin (right) concentrations, for the 10E_ANI sample. We measured fitness in each drug concentration as the empirical estimate of the area under the time-vs-growth curve (nAUC). The shaded error bars represent the 95 confidence interval of the mean across technical replicates. We used rAUC and MIC_{50} as proxies for drug susceptibility. rAUC was defined as the AUC of these data normalized by the maximum AUC (AUC_{MAX}), in which fitness is maintained across all the range of concentrations. MIC_{50} was defined as the minimum concentration where there is at least 50% of fitness inhibition, as compared to the no-drug control.

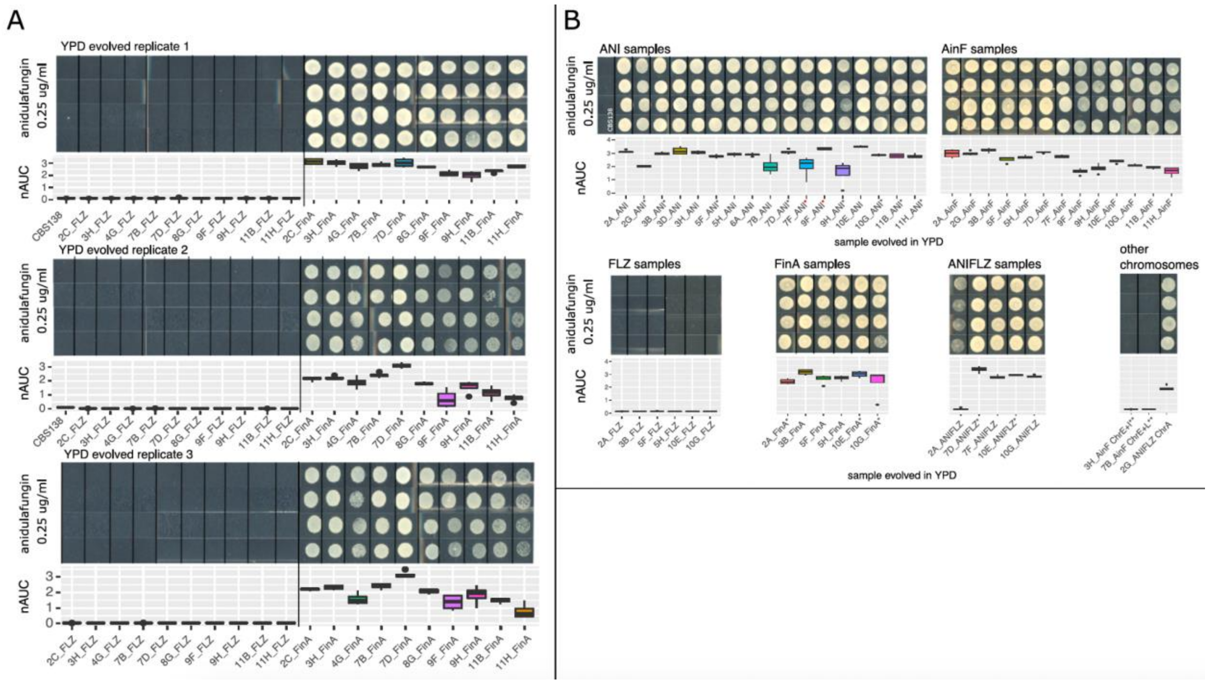

Supplementary Figure S2. Spots and areas under the growth curves (nAUC) of YPD evolved samples at 0.25ug/ml ani. Presented spots are four replicates of each evolved sample grown for 24h in the presence of the drug with corresponding areas under the growth curve. **(A)** shows AS samples, from left to right: ten FLZ_YPD and ten FinA_YPD and from top to bottom: YPD evolved replicates one to three. (**B)** 50 other tested samples divided into groups corresponding to the parental resistant samples (Ksiezopolska et al., 2021), and the three samples with other chromosomal alterations. Although the area under the growth curve in 2A_ANIFLZ_YPD sample indicates low ani resistance, the spots demonstrate the continued growth in the presence of the drug. Additionally marked are: * - parents presenting *ERG3* mutations and resistance to flz, * - parents evolved in ani presenting *ERG3* mutations and susceptibility to flz, ** - parents were not ani resistant.

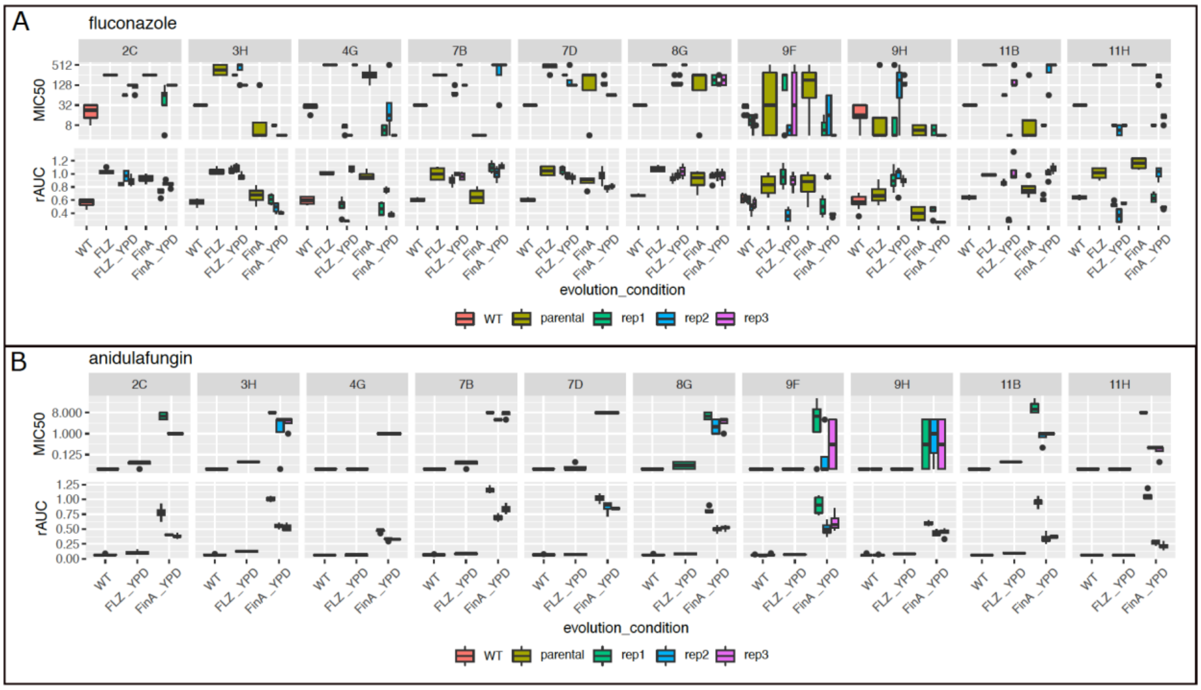

Supplementary Figure S3. Susceptibility measurements rAUC (relative Area Under the growth Curves) and MIC_{50} for AS samples grown in the presence of (A) fluconazole and (B) anidulafungin. The results are divided into ten boxes representing ten pairs of parental flz resistant (FLZ) strain and its ani-evolved progeny (FinA) where ChrE duplications appeared and were maintained, respectively. Each box indicates, in this order, the WT (unadapted strain), the FLZ resistant strain used as parental for this study, its YPD-evolved derived strain, the FinA resistant strain used as parental for this study, and its YPD-evolved derived strain. (**B**) Due to the fact that none of the progenies of ani resistant strains lost the resistance to ani (**Supplementary Figure S2**), we assessed the susceptibility to this antifungal drug in WT and YPD evolved strains only. Each box indicates, in this order, the WT, YPD-evolved (only replicate one of FLZ_YPD since FLZ parental are ani susceptible, and replicates 1-3 for FinA_YPD) AS.

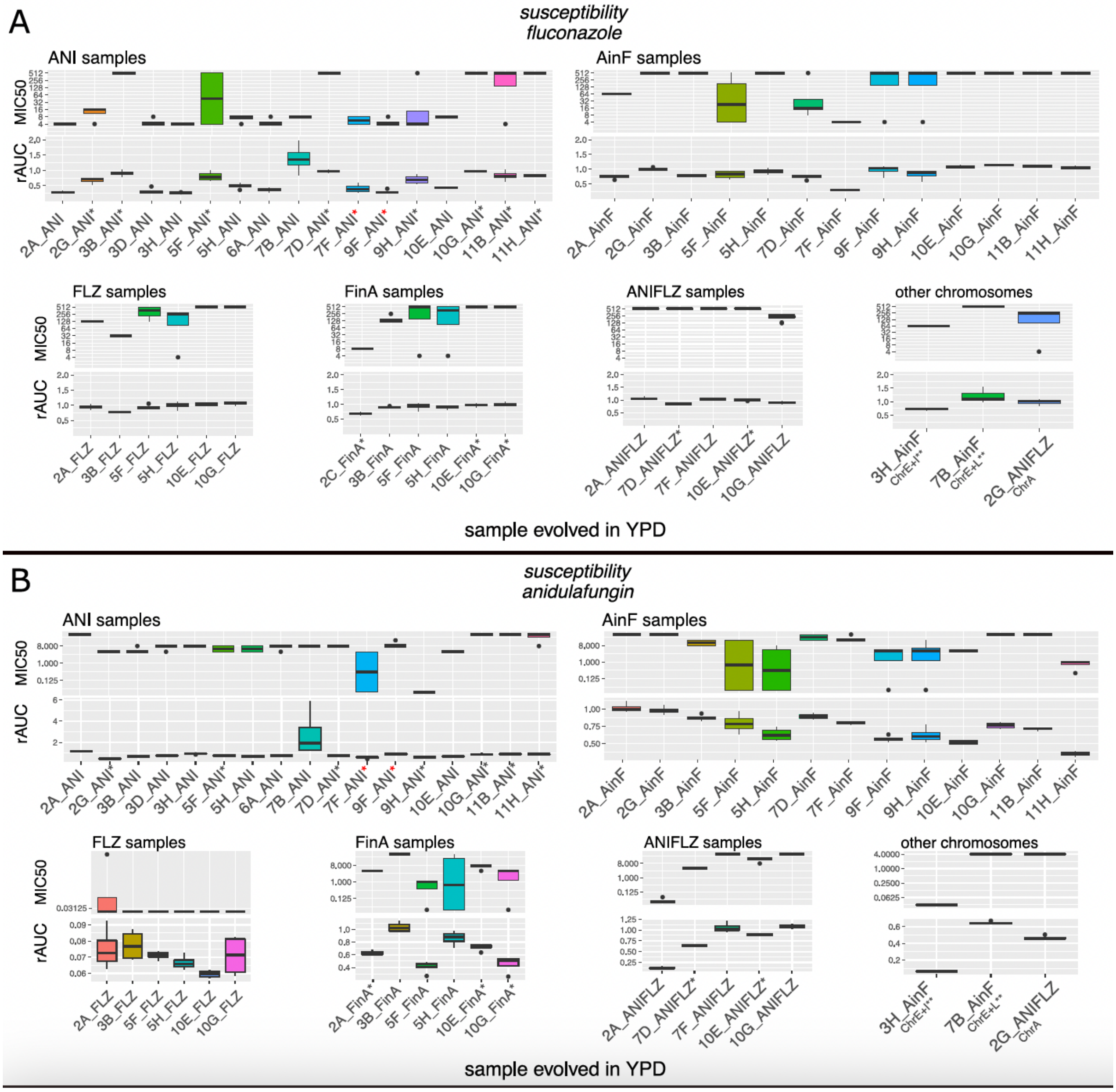

Supplementary Figure S4. Susceptibility measurements rAUC (relative Area Under the growth Curves) and MIC$_{50}$ for 50 evolved samples grown in the presence of (A) fluconazole and (B) anidulafungin. Presented are susceptibilities of YPD-evolved samples divided into groups corresponding to the parental resistant samples (Ksiezopolska et al., 2021), and the three samples with other chromosomal alterations. Additionally marked are: * - parents presenting *ERG3* mutations and resistance to fluconazole, * - parents evolved in anidulafungin presenting *ERG3* mutations and susceptibility to fluconazole, ** - parents that were not ani resistant. (**B**) Even though rAUC for ANI_YPD evolved samples seem low, all of the samples maintained the resistance (see **Supplementary Figure S2**). It seems so due to incomparably high rAUC of 7B_ANI samples connected with its low basal fitness (detailed in (Ksiezopolska et al., 2021)).

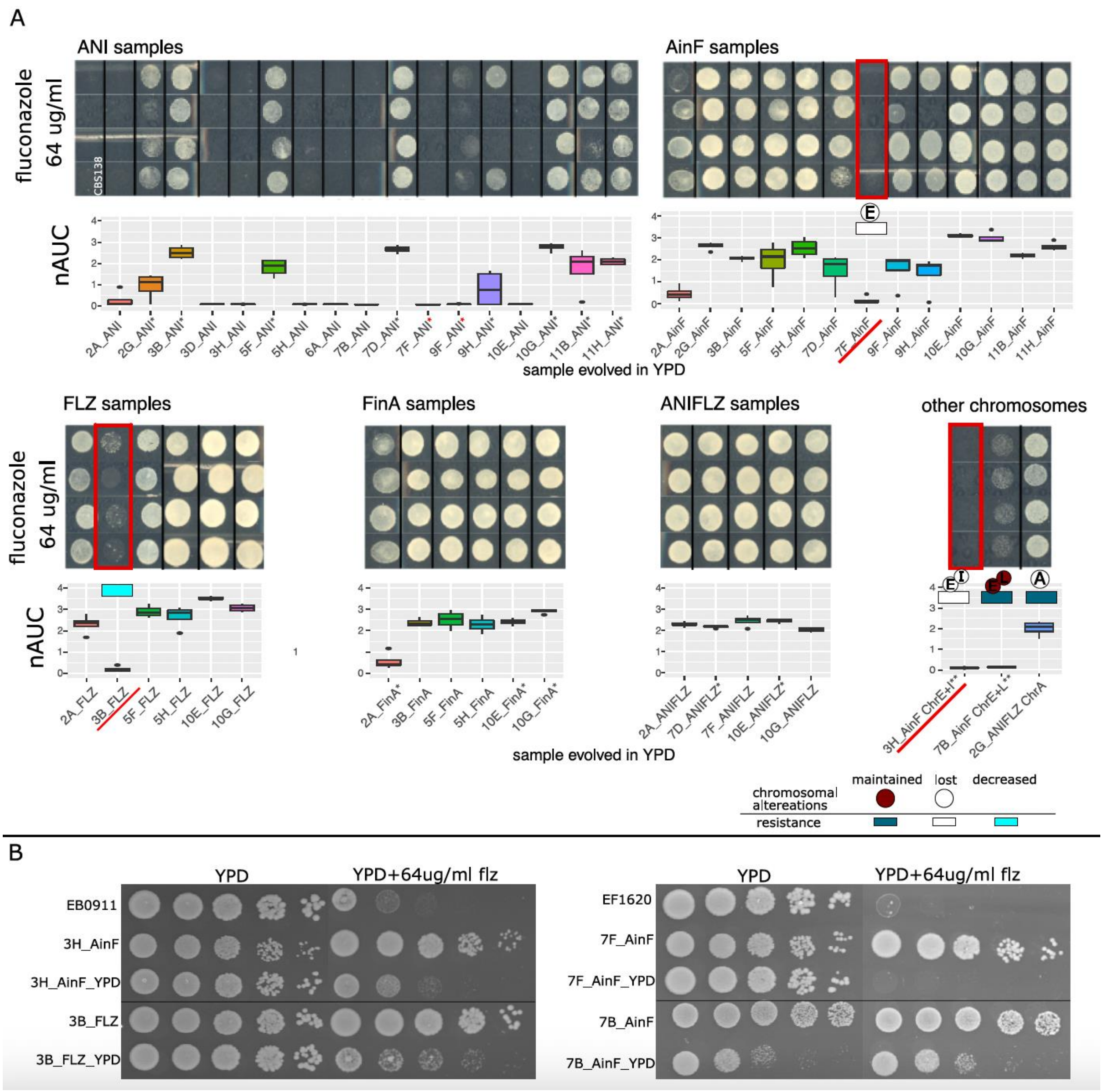

Supplementary Figure S5. (A) Spots and areas under the growth curves (nAUC) of YPD evolved samples at 64 ug/ml flz. Presented are four replicates of each sample grown for 24h in the presence of the drug divided into groups corresponding to the parental resistant samples (Ksiezopolska et al., 2021) and the three samples with other chromosomal alterations. Additionally marked are: * - parents presenting *ERG3* mutations and resistance to fluconazole, * - parents evolved in anidulafungin presenting *ERG3* mutations and susceptibility to fluconazole, ** - parents were not ani resistant and underlined samples that presented a decrease or loss of resistance to flz (**B**) **Spot test.** Additional comparison of growth on YPD and YPD supplemented with 64 ug/ml flz of samples that lost flz resistance (3H_AinF_YPD, 3B_FLZ_YPD, 7F_AinF_YPD), that showed low basal fitness (7B_AinF_YPD) and their parentals. From top to bottom presented are wild type (susceptible) strain, resistant parental and YPD evolved mutant in this study. 3B_FLZ is a good example of the partial decrease of resistance and not a full loss of the phenotype. Comparison of the spots of WT susceptible strain (EB0911), flz resistant strain (3B_FLZ) and YPD evolved strain from this study (3B_FLZ_YPD) demonstrate the different levels of the susceptibility to flz. 7B_AinF_YPD

presents a natural deficiency in fitness, even when grown at optimal (YPD) conditions. Hence, even though it seems that the sample's resistance to flz decreased (worse growth in the presence of the drug), it is apparent only due to its fitness.
